# Natural Products in Alzheimer’s Disease Therapy: Would Old Therapeutic Approaches Fix the Broken Promise of Modern Medicines?

**DOI:** 10.3390/molecules24081519

**Published:** 2019-04-17

**Authors:** Solomon Habtemariam

**Affiliations:** Pharmacognosy Research Laboratories & Herbal Analysis Services UK, University of Greenwich, Central Avenue, Chatham-Maritime, Kent ME4 4TB, UK; s.habtemariam@herbalanalysis.co.uk; Tel.: +44-208-331-8302/8424

**Keywords:** Alzheimer’s disease, cholinergic, acetylcholinesterase, amyloid beta, anti-inflammatory, antioxidant, tau protein

## Abstract

Despite extensive progress in understanding the pathology of Alzheimer’s disease (AD) over the last 50 years, clinical trials based on the amyloid–beta (Aβ) hypothesis have kept failing in late stage human trials. As a result, just four old drugs of limited clinical outcomes and numerous side effects are currently used for AD therapy. This article assesses the common pharmacological targets and therapeutic principles for current and future drugs. It also underlines the merits of natural products acting through a polytherapeutic approach over a monotherapy option of AD therapy. Multi-targeting approaches through general antioxidant and anti-inflammatory mechanisms coupled with specific receptor and/or enzyme-mediated effects in neuroprotection, neuroregeneration, and other rational perspectives of novel drug discovery are emphasized.

## 1. Introduction

**Figure 1:**
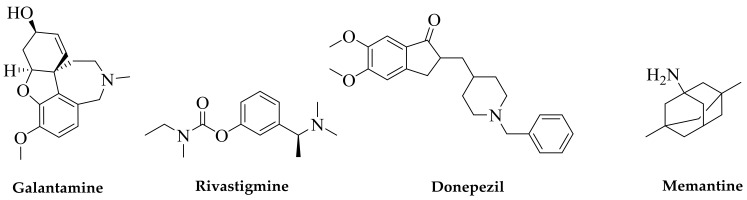
Current drugs of choice for Alzheimer’s disease (AD) therapy. Natural products are at the forefront of drug discovery for AD as galantamine was first isolated from the bulbs of the common snowdrop plant *Galanthus nivalis*. Isolated from the West African Calabar bean, physostigmine has been the prototype acetylcholinesterase (AChE) inhibitor, but its short duration of effect for AD and tacrine’s toxicity initiated the search for safer drugs; this led to the discovery of donepezil as a synthetic AChE inhibitor. Memantine acts through a different mechanism of action and was obtained from the synthesis source.

There is no other way to describe the frustration of drug discovery efforts for the treatment of Alzheimer’s disease (AD) other than by listing the number of approved drugs for its therapy. Counted by fingers on one hand, drug therapies for AD, at the very best, temporarily relieve symptoms and slow down the disease progression. Three of these approved drugs (Figure 1) are acetylcholinesterase (AChE) inhibitors (donepezil, rivastigmine, and galantamine) and the fourth is memantine which is a *N*-Methyl-d-aspartate (NMDA) antagonist. While rivastigmine and galantamine are normally prescribed for mild to moderate cases, donepezil may be used to treat all stages; and memantine, often in combination with other drugs such as donepezil, is employed for moderate to severe AD. It is interesting to note that memantine actually lacks efficacy for milder cases of AD [1,2]. The limited benefit of these drugs in some patients is associated with numerous side effects, which are all listed in the webpages of AD societies and associations, for example, [3,4]. 

Perhaps the best pathological hallmark with convincing therapeutic proof of concept for AD is based on the amyloid–beta (Aβ) hypothesis. The accumulation of Aβ that is often associated with memory impairment as well as genetic predisposition in some individuals with the Aβ precursor protein (APP), or its missense mutations that cause AD [5], have been well established. The mutations in presenilin-1 (*PSEN-1*) and presenilin-2 (*PSEN-2*) genes [6], or crosstalk between apolipoprotein E (apoE) isoforms and the encoding genes (*ApoE4*) mutation and Aβ levels [7], are further good examples of the close association between Aβ and AD; and/or the rational for Aβ targeting by therapeutic agents. Several decades of research on such therapeutic targeting by all the major pharmaceutical companies (e.g., Merck, Pfizer, J&J, Eli Lilly, and Roche), however, have led to dismal outcomes with spectacular failure rates (100%) of phase III clinical trials. It remains the case that the limited therapy we have for AD are those developed/approved during the 20 years of intense research between 1981 (tacrine) to 2001 (galantamine).

Even though our understanding of the AD pathology has greatly increased over the last 40 years, the above drug discovery reality exhibits itself upon a harsh reality of the increased disease burden over the years. According to the World Health Organization (WHO) figure [8], AD accounts for 60–70% of all cases of dementia (others include vascular dementia, dementia with Lewy bodies, etc.) and the global dementia figure in 2017 was estimated to be 50 million people with around 10 million new cases every year. With the projected dementia figure reaching 82 million in 2030 or 152 million in 2050, the social and economic cost of AD will only increase over the coming decades. The estimated global financial cost of dementia for the year 2015 and 2018 stood at US $818 billion and US $1 trillion, respectively [9]. Without losing hope for the existing late clinical trials, one must look outside the box of the current thinking if we are to make a breakthrough in AD therapy.

## 2. AD Pathology

One of the major problems associated with drug discovery efforts for AD is linked to the slow onset and progression of the disease. The most common symptoms of AD include the progressive decline in cognitive and learning/memory functions, which are also associated with behavioral abnormalities and language/speech impairments [10]. On the neuropathological side, AD is associated with neuronal loss and synaptic dysfunction in the various brain regions, but most profoundly, in areas associated with cognition. Numerous papers have put in place the neuropathological features of AD as diagnostic tools; this was elegantly presented by Serrano-Pozo et al. [11] as “positive lesions such as amyloid plaques and cerebral amyloid angiopathy, neurofibrillary tangles, and glial responses, and negative lesions such as neuronal and synaptic loss”. From a biochemical point of view, the most significant hallmark of AD is the appearance of senile plaques or aggregating Aβ and neurofibrillary tangles (NFT) that are caused by aggregating hyperphosphorylated tau proteins. In the latter case, the microtubule-associated protein tau, which is involved in the assembly and stabilization of microtubules, becomes aggregated when hyperphosphorylated by a variety of mechanisms. The failure of the Aβ therapeutic approach so far and the fact that the vast majority of AD cases do not have a genetic/familial basis still beg the question on the validity of Aβ as a therapeutic target (see the following sections). This diverse etiology may also be in part one of the reasons for the failure of clinical trials that assume AD as a single disease instead of just a common name for neurological disorders of diverse pathological origins. Considering that neuoroinflammation, oxidative stress, and excitotoxicity all play central roles in neuronal loss in AD, and they are also all induced by Aβ, abandoning the Aβ therapeutic target altogether seems to be a high-risk strategy at the moment.

## 3. The Aβ Therapeutic Approach

**Figure 2:**
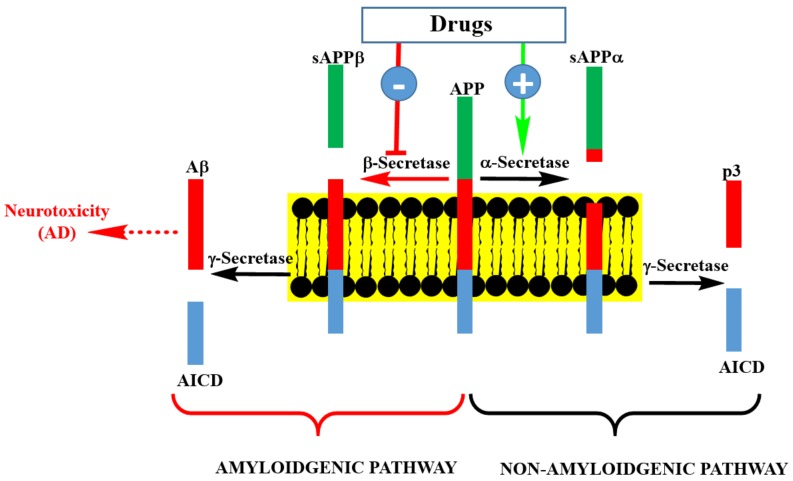
Pathways of Aβ precursor protein (APP) processing and Aβ formation. Inhibition of β-secretase by pharmacological agents is at the center of drug discovery research for AD therapy. This could be achieved by small molecular weight compounds or an antibodies approach. Enhancing the activity of γ-secretase by pharmacological agents is another possibility, though it is a less popular strategy than β-secretase inhibition. Targeting soluble or aggregated Aβ by various agents is another approach being undertaken in AD drug discovery.

The process of Aβ formation and therapeutic targets are depicted in Figure 2. APP is processed in neuronal cells through two pathways: the amyloidogenic and non-amyloidogenic pathways. The non-amyloidogenic pathway representing the normal physiological process includes the enzymatic action of α-secretase and γ-secretase, leading to the formation of peptide-soluble amyloid precursor protein α (sAPPα), amyloid precursor protein intracellular domain (AICD), and p3. On the other hand, the amyloid pathway involves the sequential cleavage of APP by β-secretase (BACE-1: β-site amyloid precursor protein cleaving enzyme 1) and γ-secretase to yield Aβ (predominantly Aβ_40_ and Aβ_42_). Theoretically, drugs that enhance the activity or expression level of α-secretase, or those downregulating the expression level of β-secretase or enzyme inhibitors could be seen as potential therapeutic agents for AD. This is also the area of frustration for the failed clinical trials of drugs such as Lanabecestat [12] and is the basis for the current pessimism in this therapeutic approach. There are still ongoing phase II/III clinical trials on BACE-1 inhibitors and perhaps we should wait to see their outcomes before making firm conclusions on the merit of this therapeutic targeting.

The Aβ monomers in the presence of an appropriate pro-aggregating environment (for example, transition metal ions) form oligomers, fibrils, β-sheets containing fibrils, and Aβ plaques. Even through Aβ plaques may be found in humans that do not display AD symptoms, or wide-spread Aβ deposits that lack β-sheet structure (or classical Aβ) may not be seen in some AD patients, the induction of neurotoxicity by Aβ under experimental conditions both in vitro and animal models is well understood. Hence, the therapeutic principle based on Aβ-aggregation inhibition or induction of disaggregation, including by metal ion chelation (copper, zinc, and iron) or their removal, have attracted a lot of therapeutic interest in recent years. While the therapeutic potential of metal chelation under a clinical setting for AD has yet to be proven, the reduction of Aβ oligomer formation by targeting these divalent cations has been well established [13,14,15]. This is in line with the role of transition metals such as iron in Aβ plaque formation and induction of neurotoxicity [16,17,18]. In view of the role of transition metals in oxidative stress-induced neuronal damage, the rationale of targeting Aβ aggregation pathways in AD appears to be feasible. Which one of the Aβ forms (soluble monomers, oligomers, fibrils, etc.) to target is still, however, a daunting task, especially given the failure of the anti-Aβ antibodies approach so far. In this direction, the very recent news in March 2019 on the failure of aducanumab in a late stage clinical trial, by another pharmaceutical giant Biogen and its Japanese partner Eisai, is another grim reminder of the risk associated with this approach. 

One further area of research that is yet to be proven valid is Aβ vaccine development. This has reached phase II and III clinical trials for some agents [19,20,21], but the most widely researched approach is immunotherapy based on Aβ antibodies. Although some good results were reported in earlier studies, lack of efficacy in phase III clinical trials have been reported for some drugs such as gantenerumab [22]. While waiting for the completion of ongoing clinical trials on this approach, the validity of such approaches is yet to be proven.

## 4. The Cholinergic Hypothesis

**Figure 3:**
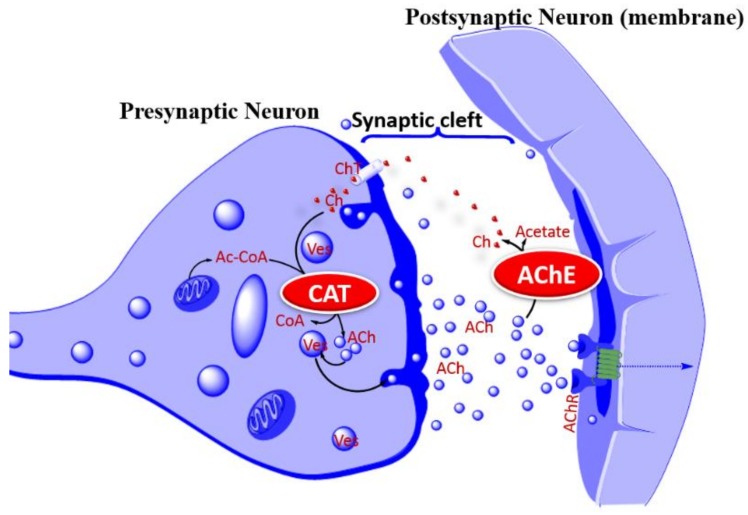
Acetylcholine release process and the cholinergic hypothesis of AD. The cholinergic hypothesis of AD is based on the putative role of cholinergic neurons in learning, memory, and cognition. Deletion of cholinergic neurons in AD is evident from the low levels of CAT, AChR, or choline in key brain regions associated with cognition. As a therapeutic proof of concept, augmenting Ach release, increasing AChR activity, or increasing the lifespan of ACh can overcome the cholinergic neuron deficiency in AD pathology. In the latter case, inhibition of AChE is the common therapeutic approach for increasing the activity of surviving cholinergic neurons. *Abbreviation:* ACh, acetylcholine; Ac-CoA, acetyl-CoA; AChE, acetylcholinesterase; AChR, acetylcholine receptor; Ch, Choline; CAT, choline acetyl transferase; CoA, coenzyme A; ChT, Choline transporter (carrier); Ves, vesicle.

Based on the role of the cholinergic (acetylcholine, ACh) system in learning and memory, and that the loss of these neurons in the basal forebrain and cerebral cortex is associated with cognitive deficits, the cholinergic hypothesis [23] of AD has been at the forefront of therapeutic approaches for AD. At the global level in the various brain regions, the deficiency of choline acetyltransferase (ChAT), the enzyme that synthesis ACh, choline uptake, or ACh release (Figure 3) are all common features to demonstrate that the deletion of these neurons is associated with learning and memory loss. In fact, the cholinergic hypothesis of memory and AD as a disorder associated with cortical neuron dysfunction were among the earliest established findings linking cholinergic neurons with dementia pathology [24,25,26,27]. Accordingly, one of the experimental models for induction of memory deficits is based on the use of scopolamine as a central nonselective, competitive, muscarinic ACh receptor-blocker [28,29]. It is not thus surprising that three of the four approved drugs are based on an approach of increasing the lifespan of ACh by inhibiting the major neuro-specific hydrolysis enzyme acetylcholinesterase (AChE). Tacrine was the first cholinesterase inhibitor to be approved for AD in 1993. With poor efficacy and an inability to alter the progression of the disease, it was discontinued in the US in 2013, mainly due to concerns over safety of systemic toxicity such as in the liver. This toxicity profile is in part linked to the long-standing debate on the required degree of selectivity of cholinesterase inhibitors between neuronal AChE and butyrylcholinesterase (BuChE) that may have other functions in the peripheral system. The hepatotoxicity of tacrine in mice in vivo [30], rat hepatocytes in primary culture in vitro [31], and alanine aminotransferase (ALT) elevation in humans [32] have been well documented. In this regard, the selectivity of donepezil to AChE, unlike tacrine, as well as kinetics of enzyme inhibition and other pharmacological effects (e.g., ACh receptors, monoamine uptake, etc.) have been under intense research. There are also many other cholinesterase inhibitors that have been tried for AD such as physostigmine or its derivatives, such as phenserine, esolerine, NS2330 (Tesofensine), or numerous synthetic inhibitors that showed pharmacological efficacies in vitro and in vivo but did not make a breakthrough as drug therapy for AD. The proof of concept for both natural and synthetic AChE inhibitors is still there, but the rationale for developing new drugs based on the same therapeutic principle when these new drugs may not be more effective than the existing approved drugs (donepezil, rivastigmine, and galantamine) has not embraced. One useful proof of concept to try is of course to combine AChE inhibition with other favorable pharmacology. In fact, tacrine analogues combining AChE inhibition with a potassium channel blockade at the nerve terminal, leading to increased ACh release/output, are a good starting point, if it was not for their unfavorable side effects. On the other hand, numerous natural products that combine AChE inhibition with a plethora of neuroprotective mechanisms have been identified (see the following sections). 

## 5. Other Neurotransmitters

Beyond memory deficits associated with the cholinergic hypothesis, AD has a hallmark of behavioral changes linked to psychological disorders such as depression. Whether these disorders contribute to the AD or just are pathological consequences are yet to be proven, but disturbances in serotonergic, noradrenergic, as well as gamma-aminobutyric acid (GABA) at some stages of cognitive dysfunction in AD have been noted [33,34]. The cross-talk between the role of serotonergic neurons in emotional behavior and cognition is evident from the findings that the serotonergic system in the hippocampus and prefrontal cortex is involved in different memory processes, spatial navigation, decision-making, working memory, attention, and reversal learning (reviewed in [35]). Similarly, other monoaminergic system including a noradrenergic neuron deficit of up to 70% in the locus coeruleus (LC) as well reduction in dopamine (along with its metabolites and receptors) have all been reported in AD (reviewed by Šimić et al. [36]). If not for the disease pathology, the symptomatic relief of AD should thus include all these other neuronal pathways that are interlinked with memory function and deficit in AD. While the role of glutamate via its ionotropic NMDA receptors in learning and memory is known, it also mediates excitotoxicity under ischemia, oxidative stress, and a range of neurodegenerative diseases such as AD. Through action both at localized synaptic and extrasynaptic sites, neuronal death induced by Ca^2+^ influx could be induced by activation of NMDA receptors [37,38,39]. The approval of memantine as an NMDA antagonist for AD therapy is in-line with this reality. All these data underpin the rationale that the design for AD drug discovery programs should consider neuroprotection and neurotransmitter approaches far beyond the scope of the cholinergic hypothesis of AD.

## 6. Tau Hyperphosphorylation and Aggregation

One of the pathological hallmarks of AD is hyperphosphorylated tau protein that aggregates into neurofibrillary tangles [40,41]. The functional integrity of tau protein is governed by the degree of its phosphorylation, and hence the balancing act of kinases and phosphatases in neuronal cells needs to be assessed when targeting NFT formation by therapeutic targets. Given the glycogen synthase kinase 3β (GSK-3β) is the major kinase enzyme for tau hyperphosphorylation, its inhibition by numerous drug candidates has been extensively studied over the last decade. There is as yet no approved drug, however, that works through inhibition of tau hyperphosphorylation and aggregation. As with the Aβ approach, the lack of efficacy under clinical studies for some of the tau aggregation inhibitors including the GSK3-β inhibitor trials have already surfaced [42,43]. The vaccine and antibody approaches are also being researched though their clinical efficacy is yet to be demonstrated. 

## 7. Neuroinflammation

Along with the intraneuronal NFT and extracellular plaques, the classical feature of AD published by Alois Alzheimer in 1907 included morphological changes in microglial cells. An English translation of this account is available [44], though a plethora of more than a century research since then has firmly established glial cells as the major modulators of the inflammatory component of neurodegenerative diseases in the CNS. One reasonable thought in the AD therapeutic approach is thus to shift from the neuronal cells themselves to induction of neuroinflammmation by the primary immune cells in the brain, glial cells, such as astrocytes, microglia, and oligodendrocytes. Stressing the genetic alteration/mutation of genes associated with AD, such as apolipoprotein E (APOE), apolipoprotein J (APOJ), and sortilin-related receptor L (SORL), that are located in glial cells, and with inflammation being an integral part of AD, the literature in this field has expanded explosively in recent years. Genetic alteration, such as that the ApoE, is also regarded as the major risk factor for developing late-onset AD [45]. 

Although the microglia play a major role in Aβ clearance, their activation may not correlate with Aβ deposition, and activated microglia may in fact contribute to Aβ pathology [46]. As shown for astroglia [47], differences in functionality of cells between human and rodents may also suggest the discrepancies of data coming from animal studies and human clinical studies in the various aspects of AD therapeutic intervention studies. The differences between murine and human microglia, which are particularly evident in aging and neurodegenerative diseases, are now also becoming evident [48,49]. Interesting insights into the differences and limitations of the current knowledge of AD based on rodent studies has been reviewed by McQuade and Blurton-Jones [50]. As with macrophages, activation of microglial cells induces the expression of proinflammatory cytokines, interleukin-1β (IL-1β), tumor necrosis factor-α (TNF-α), and IL-6. Interestingly, microglia derived from aging mice exhibit this proinflammatory profile, and microglial senescence has been long-implicated in neurodegenerative diseases [51,52]. Moreover, the correlation between proinflammatory cytokines and Aβ load in transgenic mouse models [53]; Aβ and microglial activation [54]; microglial dysfunction; and defective Aβ clearance in aging AD mice [55] have been established. As a proof of concept, an exaggerated neuroinflammation is seen as a pathological feature of normal aging and AD that may be targeted by drugs to overcome cognitive impairment.

## 8. Oxidative Stress

Mitochondrial dysfunction and high levels of reactive oxygen species (ROS) or oxidative stress are prevalent in brain regions of AD where Aβ are deposited [56,57]. Considering the association between transition metals with Aβ aggregation, induction of neurotoxicity by Aβ through ROS mechanisms has been extensively researched. These include iron [58,59], coper [60,61,62], and zinc [62,63,64]. For a variety of reasons, neuronal cells are susceptible to oxidative stress [65], and antioxidant mechanisms including superoxide dismutase (SOD) and glutathione (GSH) have been shown to decline with AD progression [66,67,68]. In fact, oxidative stress as a result of mitochondrial dysfunction is suggested to be the trigger for the pathophysiology of AD [57]. The redox active metal iron has been particularly well-studied as a source of redox-generated free radicals and in AD neurotoxicity [59,69,70,71]. Antioxidant mechanisms must therefore be included as a therapeutic strategy in AD to increase the viability of surviving neurons.

## 9. Methods of AD Efficacy Evaluations

Another interesting debate on the failure of drug trials for AD relates to the methodologies used for therapeutic efficacy evaluations. The slow onset of the disease and its slow progression means a very long period of therapeutic intervention trials before decisions on their efficacy are made. Mullane and Williams [72] elegantly presented the various animal models including over 170 transgenic rodent models, dominated by mice, for AD evaluations. The difference between the rodent and human system in AD pathology that may lead to wrong interpretations has been emphasized. Other systematic review articles, for example [73], highlight the similarities between mice and humans on microglial cells while also stressing differences in their characteristics, distribution, gene expression, and states of activation. Once again, emphasis on transgenic rodent models are coming under intense review, though both the genetic and non-genetic models of AD have been scrutinized for their strength and weaknesses [74]. Similarly, cognitive ability assessments (e.g., mini-mental state examination, Montreal cognitive assessment, Alzheimer’s disease assessment scale, etc.) in human trials that may lead to variable cognitive scores have been highlighted. While more work is required to address these issues, the numerous experimental drugs identified in animal studies with a lot of promise are simply not replicating their drug-worthiness at the clinical level. The lack of efficacy in humans thus need to be addressed through a major breakthrough in our therapeutic approaches and/or novel target identification based on more predictable experimental disease models. 

## 10. Multi-Target Approach—Lessons from Natural Products

The use of herbal mixtures or crude drugs that contain hundreds of potential active principles are normally associated with the history of mankind before the advent of modern medicine. This of course does not reflect the current use of traditional herbal medicines both in Western societies, and even more commonly in developing countries. As opposed to the monotherapy approach of many first-line modern medicines, there is no wonder why this approach of polytherapy (multidrug → multi-target → one disease) is often seen as a primitive therapeutic approach. In reality, however, polytherapy is quite common in modern medicine, especially for treating chronic diseases such as cancer or in cases where aggressive drug-combination therapy is the only alternative following monotherapy. This does not even include the concept of additive or synergistic effects of compounds/drugs, which are often involved in crude plant extracts. As with synthetic compounds, the number of crude natural products or herbs published in the scientific literature under in vitro and animal models of dementia are staggeringly large. From all the available data so far, primarily from preclinical pharmacological efficacy evidence, the therapeutic potential of crude herbal drugs for AD is clear [75]. While our chance of identifying a natural product-based drug of this nature for AD is still as good as it has ever been, there is also no evidence to suggest that we already have herbal products out there with proven efficacy for AD. Hence, the risk of taking crude plant extracts to clinical trials to find a better therapeutic agent than the existing few drugs, by and large, appears not to have been taken yet. 

The promise of identifying a better therapeutic agent for AD than the existing drugs is even more evident for the active ingredients of medicinal plants that work through multiple targets: one compound acting through multiple biological targets (one drug → multi-target → compex diseases therapeutic approach). This polypharmacology approach inherently lacks the predictability of a one-target drug entity, but its therapeutic relevance cannot be ignored any longer. Mostly being dietary-source agents, the drug molecules of natural products acting through this mechanism may be well-tolerated and could be applied in humans without a fear for their safety so long as they are proven to be efficacious. 

**Figure 4:**
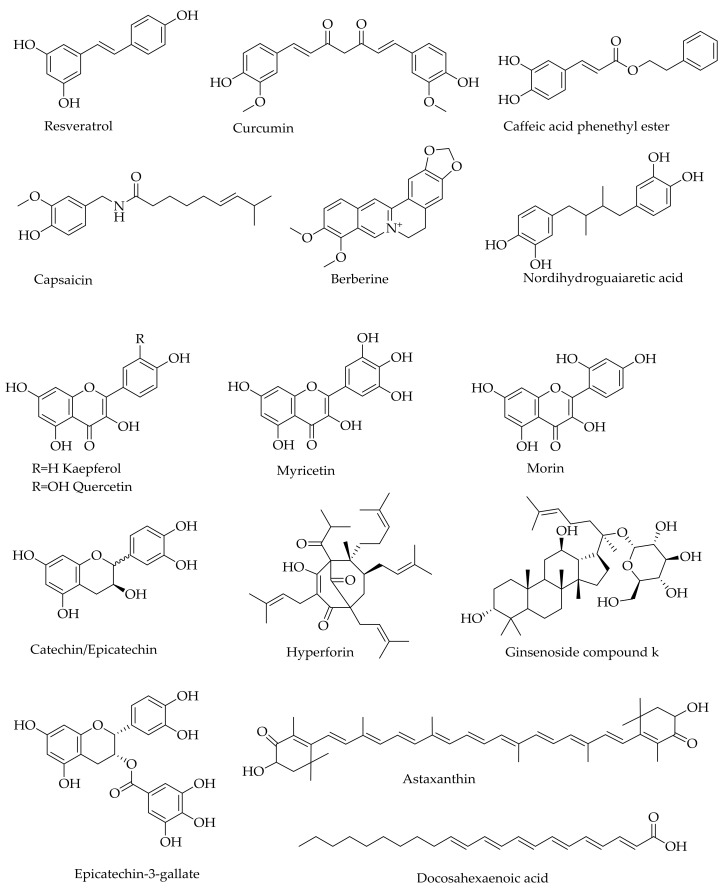
Examples of natural products that show activity against Aβ in AD experimental models.

As with the crude plant extracts, the number of natural products belonging to the various structural classes that offer neuroprotection under neurodegenerative conditions are staggeringly high [76]. Numerous review articles, including those on phenolic acids [77], flavonoids [78,79,80,81,82,83], alkaloids [83,84], monoterpenoids [85], and diterpenoids [86], provide a list with therapeutic promise for AD. The various classes of compounds that have been shown to target Aβ aggregations and prevent Aβ neurotoxicity, for example, include β-sheet-binding dyes such as Chrysamine G [87]; oligopeptides [88,89,90,91,92]; and polyphenols such as curcumin, myricetin, morin, quercetin, kaempferol (+)-catechin, (−)-epicatechin, nordihydroguaiaretic acid (Figure 4), and tannic acid [93,94,95]. While most of the identified natural products display antioxidant effects in a plethora of experimental models in vitro and in vivo, they have also been shown to ameliorate Aβ neurotoxicity through a variety of mechanisms. Even some monoterpenes that lack the basic phenolic structural moieties for direct ROS scavenging, display antioxidant effects in vivo by inducing antioxidant defenses [85]. Moreover, the activity of some monoterpenes could be enhanced by over 100-fold when other functional groups such as a carbamate moiety are added or they are incorporated into the existing anti-AD drugs such as galantamine [96,97]. As demonstrated for the active ingredients of many traditional medicines such as ginseng (e.g., ginsenoiseds and gingolides), the neuroprotective effects of such natural products through multiple mechanisms are extended to a range of diseases including ischemic stroke [98,99]. 

As a master regulator of the antioxidant mechanism (and also involved in the anti-inflammatory mechanism), the redox transcription factor, nuclear factor erythroid 2-related factor 2 (Nrf2), has emerged recently as a valid therapeutic target for AD and a number of other neurodegenerative diseases [100,101,102]. The translocation of Nrf2 to the nucleus and subsequent binding to the antioxidant response elements (ARE) in the promoter region of genes, such as heme oxygenase-1 (HO-1) and NAD(P)H:quinone oxidoreductase 1 (NQO1), has been established. Many polyfunctional natural products that have been shown to have protective effects against Aβ-toxicity, such as curcumin caffeic acid derivatives (e.g., caffeic acid phethyl ester), berberine, resveratrol, quercetin, and ginsenosides (e.g., ginsenoside compound K) (Figure 4), all induce antioxidant defenses through induction of the Nrf2/HO-1 axis [103,104,105,106,107,108].

Numerous natural compounds such as phenolics including resveratrol [109], curcumin [110], hyperforin [111], and capsaicin [112] (see Figure 4) have also been shown to display inhibitory effects against tau protein hyperphosphorylation as well as pharmacological efficacy in in vivo models of AD. Through general antioxidant and anti-inflammatory mechanisms coupled with a range of specific receptor-mediated effects, the pharmacological efficacy of such natural products in mood disorders such as depression and anxiety have further been extensively researched in recent years. In experimental models, the effect of many natural products in the established proof of concept for AD therapy is not less than that of the existing drugs. One should, at least in principle, see the therapeutic potential of such natural products that display anticholinesterase, anti-inflammatory, and antioxidant effects, such as reversing tau protein hyperphosphorylation or Aβ neurotoxicity along with numerous favorable effects including neuroprotection in experimental models of AD.

Neuroregeneration has recently emerged as a productive area of research for pathologies where neuronal cell losses are prevalent. Good examples are traumatic brain injury (TBI) and the various neurodegenerative diseases including AD. The role of neurotrophins such as the brain-derived neurotrophic factor (BDNF) in neuroregeneration has been reviewed, and many natural products that offer promise in neurodegenerative diseases also increase neuroregeneration by upregulating the expression level and/or activity of BDNF [113]. As a therapeutic target, the search for BDNF mimics [114,115] for these diseases is similar to the idea of using stem cell therapy for abrogating stroke-induced neuroinflammation and related neurodegenerative diseases [116]. Natural products of BDNF modulators include berberine, resveratrol, and epicatechin-3-*O*-gallate (Figure 4), which are all known for their multi-target pharmacology and neuroprotection in experimental AD. While the therapeutic validly of neuroregeneration induction by natural products in AD human patients is yet to be validated, new neuronal cells formation in addition to neuroprotection has a vivid therapeutic implication.

**Figure 5:**
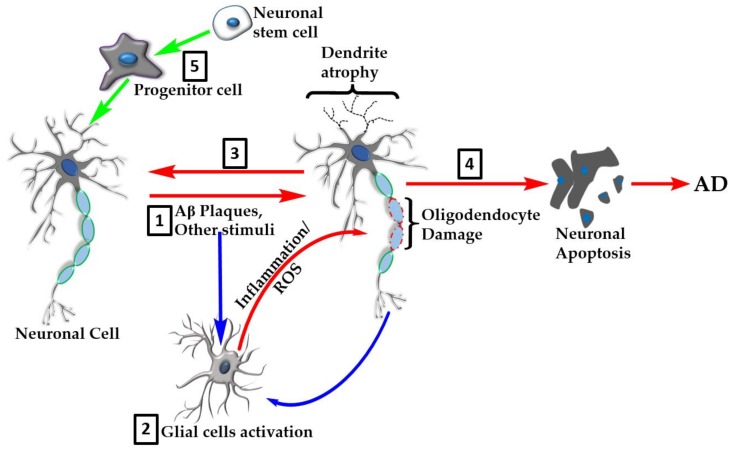
Overview of the natural products’ targets in AD. Many natural products show neuroprotective effects in the various experimental models of AD through multiple mechanisms of action. These include direct effect on neurotoxic agents such as Aβ plaque formation or tau hyperphosphorylation events (**1**). The neuroinflammation and oxidative stress linked to AD pathology is orchestrated through overactivation of glial cells (**2**) that serve as the major target for natural products. Glial cell targets include amelioration of proinflammatory cytokine production/action, removal of reactive oxygen species (ROS), and boosting of antioxidant defenses. Further effects of natural products include promotion of recovery of damaged cells (**3**) or inhibition of neuronal apoptosis (**4**). Recent evidence also shows that natural products enhance neuroregeneration by promoting stem cells proliferation and differentiation (**5**).

The extent of natural products that show promise for AD is astonishing and include the diverse group of compounds from marine resources such as red algae, sea cucumber, marine spoon worms, and many other sources [117,118,119,120]. Other natural products that have gained a lot of interest as potential modulators of AD include omega-3 fatty acids (e.g., docosahexaenoic acid) and related polyunsaturated fatty acids as well as carotrenoids such as astaxanthin (Figure 4) [121,122,123,124,125,126,127,128,129]. As with the above-mentioned phenolic compounds, these compounds also display diverse pharmacology, including amelioration of neuroinflammation and oxidative stress that are the hallmarks of AD. The overall multifunctional mechanisms of these natural products are depicted in Figure 5.

One other remarkable achievement in drug therapy scored in recent years, especially for CNS disorders, has been in making therapeutic agents more bioavailable. Although compounds such as flavonoids have been shown to display pharmacological efficacies in CNS disorders and have proven to cross the blood brain barrier, their absorption/bioavailability even from the gut is generally very poor. For curcumin, for example, over ten formulations with bioavailability data showing some degree of improvement in solubility, stability, and pharmacokinetics parameters are available in the scientific literature. Pharmacokinetic studies in human volunteer studies have also shown over 100-fold higher bioavailability for some formulations (e.g., NovaSol^®^ (185), CurcuWin^®^ (136), and LongVida^®^) [130]. Other insights into the improvement of the biological activity of polyphenolic compounds including curcumin for potential application of CNS disorders have been published [131]. Hence, with the help of new tools, even old medicines that work through polypharmacology therapeutic principles could be pushed to solve the AD therapeutic dilemma. 

## 11. Conclusions

AD is a complex disease with multiple etiologies. The current drugs of choice based on AChE inhibition or NMDA receptor antagonism offer some symptomatic relief, but do not affect the overall disease morbidly and mortality. Most of the recent drug discovery approaches have been based on the Aβ approach, which have continued to fail in late-stage clinical trials. As shown in this article, the merit of the polytherapeutic approach using natural products over monotherapy for AD has tremendous potential. The general antioxidant and anti-inflammatory mechanisms coupled with specific receptor and/or enzyme-mediated effects in neuroprotection, neuroregeneration, and other effects are worth consideration of a redirected therapeutic approach for AD. This multi-targeting approach by multifunctional natural products must be seen, however, as the beginning of an identification process that requires further optimization in potency and of the pharmacokinetics profile through further research.
